# Complex comorbidity and adherence to therapy for chronic kidney disease: disease perceptions & adherence in patients with comorbid HIV

**DOI:** 10.1186/1753-6561-9-S7-A20

**Published:** 2015-10-27

**Authors:** Marina Yostos, Christina Wyatt, Ioannis Konstantinidis

**Affiliations:** 1 Royal College of Surgeons in Ireland, Dublin, Ireland; 2 Mount Sinai Icahn School of Medicine, New York, USA

## Background

In the light of an aging HIV population and improved HIV treatment, Chronic Kidney Disease (CKD) has become a common contributor to morbidity and mortality. Despite that the management of CKD in HIV patients is a growing priority, little is known about the impact of HIV infection on CKD therapy adherence [1]. To investigate adherence to CKD medications and antiretroviral (ARV) medications in patients with co-morbid HIV infection and CKD - in order to identify modifiable predictors of dual adherence, focusing on illness representation and medication beliefs.

## Methods

This is a qualitative cross-sectional study that prospectively measures the relationship between disease perceptions and adherence using self-report instruments and the electronic Medication Event Monitoring System (MEMS).

HIV viral loads were used as surrogate markers to validate self-reported adherence to ARV medications. Depression, self-efficacy, alcohol and substance abuse and health literacy were assessed [2]. 20 well-characterized patients with CKD/ESRD and HIV were recruited. Institutional Review Board (IRB) approval was granted. Consent and confidentiality were protected under the Health Insurance Portability and Accountability Act (HIPAA).

## Results

(I) Demographics Data: Gender, language, ethnicity, race, education, income marital status and disease precedence did not affect adherence. Higher comorbidity and pill burden correlated with dual non-adherence. (II) Adherence Data: 65% of participants reported dual adherence, 15% reported mono-adherence and 20% reported non-adherence. ARV self-reported adherence correlated with viral load and CD4+ counts. (III) Barriers & Facilitators of Adherence: Higher self-efficacy, care satisfaction and trust in physician correlated with adherence. Depression, smoking, psychiatric illness, alcohol or substance abuse correlated with dual non-adherence. (IV) HIV & CKD Illness Perceptions: Negative disease perceptions such as stigma and guilt correlated with non-adherence. (V) Medication Beliefs: Negative medication beliefs were more prevalent among ARV non-adherent patients. Adherence was higher in patients that reported better understanding of disease and medication.

## Conclusion

The majority of participants perceived CKD to be their main health concern possibly because CKD improvement requires a combination of lifestyle modifications and medication adherence [3].
